# Decreased Intrinsic Functional Connectivity in First-Episode, Drug-Naive Adolescents With Generalized Anxiety Disorder

**DOI:** 10.3389/fnhum.2018.00539

**Published:** 2019-01-11

**Authors:** Fan Yang, Linlin Fan, Tianyi Zhai, Ying Lin, Yuyin Wang, Junji Ma, Mei Liao, Yan Zhang, Lingjiang Li, Linyan Su, Zhengjia Dai

**Affiliations:** ^1^Guangdong Mental Health Center, Guangdong General Hospital, Guangdong Academy of Medical Sciences, Guangzhou, China; ^2^Department of Psychology, Sun Yat-sen University, Guangzhou, China; ^3^The Affiliated Brain Hospital of Guangzhou Medical University (Guangzhou Huiai Hospital), Guangzhou, China; ^4^Department of Psychiatry, The Second Xiangya Hospital of Central South University, Changsha, China

**Keywords:** generalized anxiety disorder, connectome, functional connectivity, graph theory, supramarginal gyrus, superior parietal gyrus

## Abstract

Generalized anxiety disorder (GAD) is characterized by excessive and uncontrollable worry about everyday life. Prior neuroimaging studies have demonstrated that GAD is associated with disruptions in specific brain regions; however, little is known about the global functional connectivity maps in adolescents with GAD. Here, first-episode, medication-naive, adolescent GAD patients (*N* = 36) and healthy controls (*N* = 28) (HCs) underwent resting-state functional MRI (R-fMRI) and completed a package of questionnaires to assess clinical symptoms. Functional connectivity strength and seed-based functional connectivity were employed to investigate the functional connectivity architecture. GAD patients showed reduced functional connectivity strength in right supramarginal gyrus (SMG) and right superior parietal gyrus (SPG) compared with HCs. Further seed-based functional connectivity analysis revealed that GAD patients displayed decreased functional connectivity between right SMG and left fusiform gyrus, inferior temporal gyrus, parahippocampal gyrus, bilateral precuneus and cuneus, and between right SPG and bilateral supplementary motor area and middle cingulate gyrus, as well as between the SMG-based network and the SPG-based network. Moreover, the disrupted intra-network connectivity (i.e., the SMG-based network and the SPG-based network) and inter-network connectivity between the SMG-based network and the SPG-based network accounted for 25.5% variance of the State and Trait Anxiety Inventory (STAI) and 39.5% variance of the trait subscale of STAI. Our findings highlight the abnormal functional architecture in the SMG-based network and the SPG-based network in GAD, providing novel insights into the pathological mechanisms of this disorder.

## Introduction

Generalized anxiety disorder (GAD) is a common anxiety disorder characterized by excessive and uncontrollable worry about various aspects of life, accompanied by somatic symptoms including muscle tension, fatigue, and sleep disturbance (American Psychiatric Association, 2013). Given its non-invasive nature and relatively high spatial resolution compared with other functional neuroimage technologies (e.g., electroencephalography, magnetoencephalography), resting-state functional MRI (R-fMRI) has been extensively applied to investigations of brain connectivity in individuals from both community and clinical settings (Biswal et al., 1995; Kelly et al., 2012), including individuals with GAD. Prior R-fMRI research in GAD mainly focused on local functional abnormalities in certain predefined regions (e.g., amygdala, hippocampus) (Etkin et al., 2009; Chen and Etkin, 2013) and functional connectivity (FC) disruptions, including FC between amygdala and prefrontal cortex (PFC) (Etkin et al., 2009; Liu et al., 2015), posterior cingulate cortex (PCC) (Strawn et al., 2012), ventral cingulate cortex (Etkin and Schatzberg, 2011), temporal pole (Li et al., 2016), between medial prefrontal cortex (mPFC) and PCC (Andreescu et al., 2014), and between hippocampus and fusiform (Cui et al., 2016).

In addition to alterations in specific regions and FC, a review by Sylvester et al. (2012) suggests that patients with GAD display extensive disruptions in several subnetworks (e.g., default mode network, fronto-parietal network). Recently, combinations of R-fMRI and graph theory offer a new way to reveal detailed and comprehensive FC information across the whole brain (Bullmore and Sporns, 2009; Kelly et al., 2012). This approach is conducive to examining the interactions of the brain in a general state (Biswal et al., 2010; Cole et al., 2010). However, whole-brain functional connectome abnormalities of GAD using graph metrics still remains unclear. Identifying such alterations can shed light on the pathological mechanisms that may underlie the multi-domain disruptions of GAD (e.g., emotion, cognition, and somatosensory), which may further provide novel insights and potential biomarkers for clinical prevention of, and interventions for, this disorder.

In the present study, we used R-fMRI and voxel-based graph theory analysis to investigate abnormal brain connectivity in first-episode, medication-naive, adolescent GAD patients without comorbidity in comparison with healthy controls (HCs). The voxel-based analysis used here avoids potential topological changes due to different parcellation approaches (de Reus and van den Heuvel, 2013). We aimed at determining the comprehensive FC disruptions among adolescents with GAD, and whether such alterations could be associated with clinical characteristics in GAD.

## Materials and Methods

### Participants

Initially, 1885 participants were recruited via advertisement and school posters from October 2011 to July 2012, and were assessed using the Screen for Child Anxiety Related Emotional Disorders (SCARED) as described in our previous study (Liao et al., 2014). Written informed consents were obtained from parents or legal guardians of all participants in accordance with the Declaration of Helsinki. This research protocol was approved by the local Medical Ethics Committee in the Second Xiangya Hospital of Central South University, China. Individuals with a total score higher than 25 were recognized as adolescents with anxiety disorders (Birmaher et al., 1999; Su et al., 2008). Accordingly, we found that there were 508 participants with SCARED scores ≥25, and the rest had SCARED scores <25. Then, 673 participants, including 508 adolescents with SCARED score ≥25, and 165 adolescents with SCARED scores <25 (randomly selected from the below-cutoff group) were interviewed and diagnosed by trained clinicians to establish the final GAD patient and HCs groups. The diagnosis protocol was established by the semi-structured instrument, Schedule for Affective Disorders and Schizophrenia for School Age Children-Present and Lifetime version (K-SADS-PL), which was conducted independently by one certified pediatric psychiatrist according to the DSM-IV criteria (Kaufman et al., 1997). Inclusion criteria for GAD patients were: (1) current first-episode, medication-naive of GAD; (2) without comorbidity; and (3) between the ages of 13–18 years old. HCs had no personal or family history of psychosis. Exclusion criteria included pervasive developmental disorder, mental retardation, Tourette’s syndrome, conduct disorder, bipolar disorder, mania, current major depression disorder, other kinds of anxiety disorders, psychotic disorder, history of head injury or seizures, and alcohol and substance abuse. Finally, 36 adolescents with GAD (mean age = 16.9 ± 0.6 years) and 28 age- and sex-matched HCs (mean age = 16.5 ± 0.9 years) were included in the current study (see Table 1). This dataset has been previously used to examine dynamic and frequency-specific FC in GAD patients (Yao et al., 2017; Zhang et al., 2017).

**Table 1:** Demographic and clinical variables for GAD and HCs.

	GAD (*N* = 34)	HCs (*N* = 26)	*P-*value	Cohen’s *d*
Age (years)	16.9 (0.6)	16.5 (0.9)	0.148^e^	0.52
IQ	102 (8.2)	106.7 (9.0)	0.081^e^	–0.53
Sex (female/male)	18/16	12/14	0.602^f^	/
PSWQ	55.4 (9.3)	38.8 (10.9)	<0.001^e^	1.62
STAI (total)	95.5 (12.0)^a^	88.0 (14.3)^c^	0.039^e^	0.56
STAI (trait)	52.2 (7.4)^b^	45.8 (9.7)^d^	0.006^e^	0.74
STAI (state)	43.7 (7.1)^a^	41.6 (7.2)^d^	0.295^e^	0.29

*Values represented mean (standard deviation) or the number of participants. GAD, generalized anxiety disorder; HCs, healthy controls; PSWQ, Penn State Worry Questionnaire; STAI (total), total score of State and Trait Anxiety Inventory; STAI (trait), the trait anxiety subscale score of State and Trait Anxiety Inventory; STAI (state), the state anxiety subscale score of State and Trait Anxiety Inventory. ^*a*^32 participants completed the questionnaire. ^*b*^33 participants completed the questionnaire. ^*c*^22 participants completed the questionnaire. ^*d*^24 participants completed the questionnaire. ^*e*^Using two-sample *t*-test. ^*f*^Using Pearson Chi-square test.*

### Clinical Assessment

Clinical symptoms were collected by administrating the Penn State Worry Questionnaire (PSWQ) and the State and Trait Anxiety Inventory (STAI) on the day of scanning. All participants were right handedness and had normal full scale IQ (>80) as measured by the Wechsler Abbreviated Scale of Intelligence (WASI).

### MRI Acquisition and Preprocessing

MRI imaging data were acquired using a Philips 3.0 Tesla scanner, equipped with a SENSE-8 channel head coil. Participants were instructed to relax, keep their heads still, eyes closed, and think of nothing during the MRI scanning procedure. The resting state functional images were obtained using gradient recalled echo-echo planar imaging (GRE-EPI) with the following parameters: repetition time (TR) = 3000 ms; echo time (TE) = 30 ms; flip angle = 90°; slice thickness = 4 mm; field of view (FOV) = 240 mm × 240 mm; 36 *trans*-axial slices with no gap. The scan lasted for 540 s. T1-weighted data were obtained using 3D rapid acquisition gradient echo sequence with the following parameters: TR = 7.5 ms; TE = 3.7 ms; flip angle = 8°; FOV = 256 mm × 256 mm; slice number = 180; voxel size = 1 mm × 1 mm × 1 mm; axial slices.

R-fMRI images were preprocessed using Statistical Parametric Mapping (SPM12^1^) and Data Processing Assistant for Resting-State fMRI (DPARSF) (Yan and Zang, 2010). The preprocessing procedure involves slice-timing, realignment, coregistration using a T1-weighted structural image, normalization to MNI space, smoothing (FWHM = 4 mm), detrend, filtering (0.01–0.08 Hz), and nuisance regression (including Friston 24 head motion parameters, white matter, and cerebrospinal fluid signal). Four participants (two from each group) were excluded due to excessive head motion (>3 mm in displacement or 3° in rotation).

### Functional Connectivity Analyses

To explore the effects of GAD on FC, Pearson’s correlations were performed for each participant between the time series of every pair of voxels within a gray matter (GM) mask (*N* = 63033) to yield a whole-brain FC matrix. The GM mask was generated by setting a threshold (cutoff = 0.2) on the mean GM probability map of all participants and with non-zeros standard deviations of blood oxygen level-dependent (BOLD) time series. Next, to improve normality, individual functional connectivity matrixes were converted to *Z*-scores with Fisher’s Z-transformation. Then, a correlation threshold was used to eliminate weak correlations possibly caused by signal noise. Since previous studies showed that results were independent of different correlation thresholds (Buckner et al., 2009; Dai et al., 2015; Liu et al., 2016), we used 0.2 as the threshold here. Because of the obscure explanation of negative correlations (Murphy et al., 2009), our analyses were only performed on positive correlations. Finally, functional connectivity strength (FCS) was calculated as the average weight of the connections between a certain voxel and all the other voxels (Dai et al., 2015; Liu et al., 2016). Notably, FCS resembles the weighted degree centrality of a network in the graph theory and reflects the global information communication ability of the brain regions (Rubinov and Sporns, 2010).

Furthermore, to examine the detailed connectivity changes of the regions with significant GAD-related FCS alterations, seed-based FC analysis was performed. This analysis takes a step further from the rough identification of the regions with abnormal information exchange ability (i.e., FCS) to specific description of their disrupted whole-brain FC patterns. Seed regions of interest (ROI) were defined as 6-mm radius spheres centered on the peak MNI coordinates of clusters that displayed significant between-group differences in FCS. For each seed ROI, a whole-brain FC map was calculated by correlating the mean BOLD time series of the given seed ROI with all the other voxels within the GM mask. Finally, the resulting connectivity maps were converted using Fisher’s Z-transformation. These result in two circuits or networks formed by the two seeds with significant between-group differences in FCS [i.e., right supramarginal gyrus (SMG) and superior parietal gyrus (SPG), see section “FCS Mapping” for more details]. To further quantify the relationship between network-level properties and clinical symptoms in the GAD group, we computed two additional network measures, namely intra-network connectivity and inter-network connectivity. The intra-network connectivity was represented by the mean peak FC values of the clusters with significant between-group differences in the seed-based FC analysis using right SMG and SPG as the seeds, whereas the inter-network connectivity was calculated by the Pearson’s correlations between the time series of the clusters with significant between-group differences in the seed-based FC analysis (including the two seeds).

### Statistical Analyses

Age, IQ, and all clinical variables were compared using two-sample *t*-tests, while sex was contrasted using Pearson Chi-square tests. To investigate the differences in connectivity measures between GAD patients and HCs, voxel-wise general linear models (GLM) were conducted to compare FC maps with age, IQ, sex, and framewise displacement (FD; see section “Head Motion Effects”) as covariates. One-sample *t*-tests were first conducted on the FC maps in each group to constrain subsequent analyses on significant positive connections (Etkin et al., 2009; Roy et al., 2013). Then, the GLM analysis was performed in the mask combining the positive FC maps of GAD and HCs group. We initially set the significance threshold of between-group differences for FCS at *p* < 0.05, combined with an individual voxel threshold of *p* < 0.001 and a cluster size >35 voxels. However, no significant cluster survived. Given the exploratory nature of calculating whole-brain FCS to search for potential seed ROIs, the significance threshold of the FCS was adjusted to *p* < 0.01 with a cluster size of 113 voxels (corresponding to a corrected *p* < 0.05). For FC maps, the significance threshold was set at *p* < 0.001 with a cluster size of 48/50 voxels (the number of voxels varied for different seed ROIs, all corresponding to a corrected *p* < 0.01). This correction was based on the Monte Carlo simulations in the Data Processing and Analysis for Brain Imaging (DPABI) toolkit (V2.3_170105) (Yan et al., 2016), which adapted the AlphaSim program in the Analysis of Functional NeuroImages (AFNI) (Cox, 2012). DPABI is a powerful toolkit that invokes and improves the functions in SPM, AFNI, and Resting-State fMRI Data Analysis Toolkit (REST) (Song et al., 2011), and has been widely used in R-fMRI studies (Takeuchi et al., 2017; Cavedo et al., 2018; Hernández et al., 2018). We also calculated Cohen’s *d* for the mean FCS and FC within the clusters to reveal the effect sizes of the observed between-group differences.

To explore the relationship between the abnormal functional connectivity and clinical symptoms, partial correlation (with age, IQ, sex, and FD controlled) was conducted in the GAD patient group between clinical measures [i.e., PSWQ, STAI (total), STAI (trait), STAI (state)] and intra-network connectivity and inter-network connectivity. We also computed four linear regression models in the GAD patient group to determine how much functional connectivity contributed to diseased-related behaviors. Three independent variables (intra-network connectivity of the SMG-based network, intra-network connectivity of the SPG-based network, and inter-network connectivity between the SMG-based network and the SPG-based network, see section “Seed-Based Functional Connectivity Mapping” for more details) were entered into the models. The significance of the changes of variance (ΔR^2^), adjusted variance (R^2^), and the standardized regression coefficient of each predictor was examined. Visualization in the current study was performed via BrainNet Viewer^2^ (Xia et al., 2013).

### Head Motion Effects

Recent studies have documented that head motion has confounding effects on functional connectivity analysis (Power et al., 2012; Satterthwaite et al., 2012; Van Dijk et al., 2012). To further reduce the confounding effects of head motion after regressing out Friston 24 head motion parameters, we employed two strategies: (1) we computed the FD of Jenkinson (Jenkinson et al., 2002) in both groups and treated mean FD as a covariate in further analyses; (2) we performed scrubbing. We deleted preprocessed volumes with FD > 0.5 mm (Power et al., 2012), as well as the previous volume and the two following volumes, and replaced the discarded volumes using the linear interpolation approach to keep the same length of time series for each participant. After that, we re-performed the FCS and seed-based FC analyses to evaluate whether our main results were influenced by head motion. The significance threshold of between-group differences was set at *p* < 0.01 with a cluster size of 110 voxels for FCS (corresponding to a corrected *p* < 0.05), and at *p* < 0.001 with a cluster size of 47–53 voxels for FC maps (the number of voxels varied for different seed ROIs, all corresponding to a corrected *p* < 0.01).

## Results

### Demographic and Clinical Variables

Demographics and clinical characteristics are summarized in Table 1. There was no significant difference between the GAD patient and HCs groups in terms of age, IQ and sex (all *p*s > 0.05). As expected, the GAD patient group showed higher scores in the PSWQ (*p* < 0.001), STAI (*p* = 0.039), and the trait anxiety subscale of STAI (*p* = 0.006) when compared to HCs, confirming that the GAD patient group was more anxious than the HCs group. The effect sizes are also shown in Table 1.

### FCS Mapping

**FIGURE 1:**
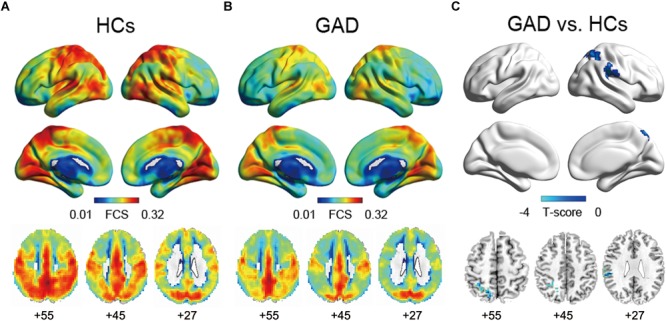
Functional connectivity strength maps of the HCs group **(A)**, the GAD patient group **(B)**, and between-group differences **(C)**. The significance threshold was set at *p* < 0.01 with cluster size of 113 voxels, corresponding to a corrected *p* < 0.05. HCs, healthy controls; GAD, generalized anxiety disorder.

In both the HCs and GAD patient groups, regions with high FCS were mainly distributed in parietal cortices (e.g., inferior parietal lobe, precuneus) and temporal cortices (e.g., inferior and middle temporal lobe) (Figures 1A,B), which was consistent with previous studies (Buckner et al., 2009; Liang et al., 2013; Dai et al., 2015). Between-group comparisons revealed that patients with GAD showed decreased FCS in right SMG and SPG (*p* < 0.05, corrected, Figure 1C and Table 2). The effect size of each cluster is also demonstrated in Table 2.

**Table 2:** Regions showing FCS differences between GAD and HCs.

Brain regions	BA	Volume (mm^3^)	MNI coordinates (x, y, z)			*T*-score	Cohen’s *d*
Right SMG	40	3321	51	–30	27	–3.81	–0.96
Right SPG	7	3105	18	–63	51	–3.65	–0.92

*GAD, generalized anxiety disorder; HCs, healthy controls; BA, Brodmann’s area; x, y, z, coordinates of primary peak locations in the MNI space; SMG, supramarginal gyrus; SPG, superior parietal gyrus. *p* < 0.05, corrected for multiple comparisons.*

### Seed-Based Functional Connectivity Mapping

**FIGURE 2:**
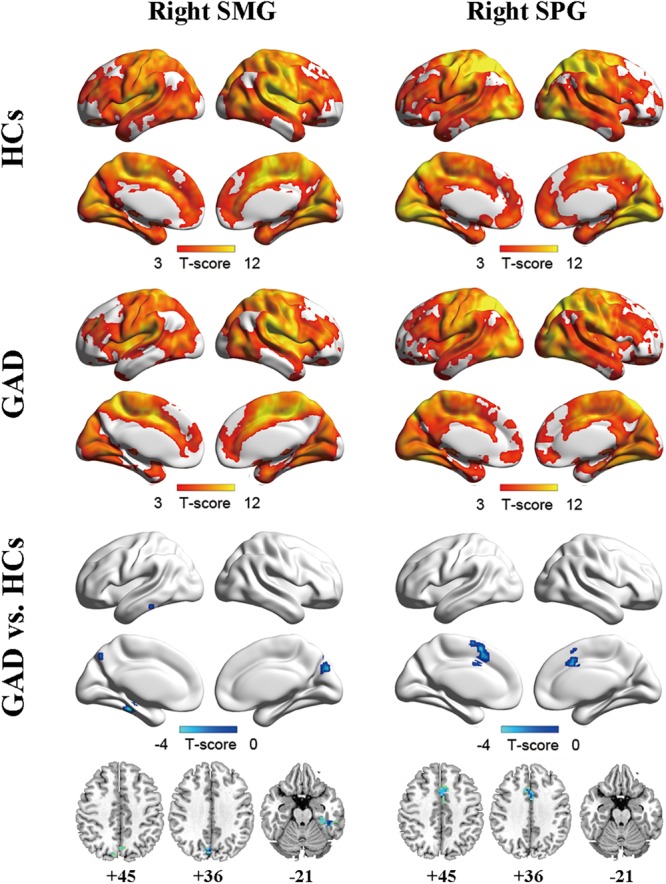
Functional connectivity maps for right SMG **(left column)** and right SPG **(right column)** of the HCs group **(upper)**, the GAD patient group **(middle)**, and between-group differences **(lower)**. The significance threshold was set at *p* < 0.001 with cluster size of 48 voxels for right SMG as ROI and 50 voxels for right SPG as ROI, corresponding to a corrected *p* < 0.01. HCs, healthy controls; GAD, generalized anxiety disorder; SMG, supramarginal gyrus; SPG, superior parietal gyrus.

Two ROIs (i.e., SMG, SPG) were derived from the regions exhibiting significant between-group differences in FCS (Figure 1C and Table 2). Results of one-sample *t*-tests and between-group differences of functional connectivity of each seed ROI were shown in Figure 2 and Table 3. Generally, voxel-based FC of each seed ROI was significantly reduced in GAD patients. Between-group comparison analysis revealed that GAD patients displayed reduced FC between right SMG and left fusiform gyrus (FFG), inferior temporal gyrus (ITG), parahippocampal gyrus (PHG), bilateral precuneus and cuneus (*p* < 0.01, corrected, Figure 2 and Table 3). We also observed decreased FC between right SPG and bilateral middle cingulate gyrus (MCG) and supplementary motor area (SMA) in the GAD patient group (*p* < 0.01, corrected, Figure 2 and Table 3). The Cohen’s *d* values were also reported in Table 3. The intra-network connectivities of the SMG-based network [*t*(58) = -5.10, *p* < 0.001, Cohen’s *d* = -1.30] and the SPG-based network [*t*(58) = -4.42, *p* < 0.001, Cohen’s *d* = -1.10] were reduced in GAD patients. More importantly, the inter-network connectivity between the SMG-based network and the SPG-based network also decreased significantly in the GAD patient group [*t*(58) = -3.294, *p* = 0.002, Cohen’s *d* = 0.85].

**Table 3:** Regions showing FC differences between GAD and HCs.

Brain regions	BA	Volume (mm^3^)	MNI coordinates (x, y, z)			*T*-score	Cohen’s *d*
**Seed: right SMG**							
Left FFG/ITG/PHG	20	1404	–45	–36	–21	–4.47	–1.12
PCu/Cu	7/19	1536	3	–78	36	–4.33	–1.08
**Seed: right SPG**							
SMA/MCG	6/24/32	4293	3	12	39	–4.42	–1.10

*GAD, generalized anxiety disorder; HCs, healthy controls; BA, Brodmann’s area; x, y, z, coordinates of primary peak locations in the MNI space; SMG, supramarginal gyrus; FFG, fusiform gyrus; ITG, inferior temporal gyrus; PHG, parahippocampal gyrus; PCu, precuneus; Cu, cuneus; SPG, superior parietal gyrus; SMA, supplementary motor area; MCG, middle cingulate gyrus. *p* < 0.01, corrected for multiple comparisons.*

### The Relationship Between Functional Connectivity and Clinical Symptoms

**FIGURE 3:**
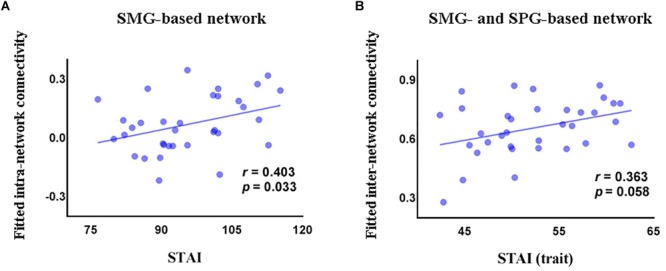
Correlations (partial correlation) in the GAD patients between STAI and intra-network connectivity of the SMG-based network **(A)**, and between the trait subscore of STAI and inter-network connectivity between the SMG-based network and the SPG-based network **(B)**. STAI, State and Trait Anxiety Inventory; STAI (trait), the trait anxiety subscale score of State and Trait Anxiety Inventory; SMG, supramarginal gyrus; SPG, superior parietal gyrus.

In the GAD patient group, we observed a significant positive correlation between intra-network connectivity of the SMG-based network and STAI (*r* = 0.403, *p* = 0.033, Figure 3A), and a marginally significant positive correlation between the inter-network connectivity between the SMG- and the SPG-based networks and the trait subscale of STAI (*r* = 0.363, *p* = 0.058, Figure 3B).

Linear regression analysis revealed that FC could predict STAI (Δ*R*^2^ = 0.242, *F*(7,24) = 2.516, *p* = 0.043), explaining 25.5% of the variance. In this model, the regression coefficient of intra-network connectivity of the SMG-based network, intra-network connectivity of the SPG-based network, and inter-network connectivity between the SMG- and SPG-based networks was 0.239 (*p* = 0.227), -0.444 (*p* = 0.061), and 0.356 (*p* = 0.127), respectively. Functional connectivity also predicted the trait subscale of STAI (Δ*R*^2^ = 0.182, *F*(7,25) = 3.984, *p* = 0.005), explaining 39.5% of the variance. The regression coefficient of intra-network connectivity of the SMG-based network, intra-network connectivity of the SPG-based network, and inter-network connectivity between the SMG- and the SPG-based networks was 0.143 (*p* = 0.415), -0.343 (*p* = 0.101), and 0.408 (*p* = 0.055), respectively.

### The Effects of Head Motion

Across all participants, the mean FD was 0.14 mm. No significant difference was found in the mean FD between GAD patients and HCs (*p* = 0.806). We also observed insignificant correlations between FD and clinical symptoms as well as neuroimaging measures (all *p*s > 0.05). In the validation analyses with scrubbing, FCS patterns of the GAD patient group, the HCs group, and the between-group differences (Supplementary Figures S1A–C) were similar to our main results (Figures 1A–C). Specifically, three clusters survived in the comparison between GAD patients and HCs, including right SMG, right SPG, and left inferior parietal gyrus (IPG). More importantly, decreased FC maps of right SMG and SPG were also found in the GAD patients (Supplementary Figures S1D–F), which were compatible with our main results without scrubbing.

## Discussion

In the current study, we used resting-state fMRI to examine voxel-wise functional connectivity alterations in adolescents with GAD. We found that adolescents with GAD showed decreased FCS in right SMG and SPG compared with adolescents in the HCs group. GAD patients also displayed reduced FC within the SMG-based network, the SPG-based network, and between the SMG-based network and SPG-based network. The effect sizes of these observed GAD-related functional connectivity abnormality were relatively large (Fritz et al., 2012). The disrupted intra-network connectivity and inter-network connectivity were correlated with clinical measures of GAD patients, and explained 25.5–39.5% of the variance of clinical symptoms. Notably, GAD usually begins in adolescence (Costello et al., 2003; Merikangas et al., 2010), and early onset (e.g., in adolescence) of GAD was associated with an indicator of adulthood depression (Copeland et al., 2012), highlighting the importance of investigating the pathology of adolescent GAD.

Areas with significantly reduced FCS in GAD patients were mainly distributed in the parietal cortex, including the right SMG and SPG. Prior studies suggested that the SMG is involved in integrating multiple sensory signals (Lopez and Blanke, 2011; Zu Eulenburg et al., 2012), overcoming emotional egocentricity (Silani et al., 2013), and processing communicative intentions (Enrici et al., 2011), which are crucial to social perception. Since GAD patients tend to perceive ambiguous social situations as negative, reduced FCS in SMG may be a potential neural substrate of inaccurate sensory integration and interpretation of social stimuli, which may lead to more vulnerability to negative emotions (e.g., anxiety). Moreover, we also found reduced functional connectivity in the SMG-based network in GAD patients, including bilateral precuneus, cuneus, left FFG, ITG, and PHG. These findings are in line with previous studies that reported decreased functional connectivity in a network formed by SMG, FFG, PHG, and precuneus in individuals with high trait anxiety (Modi et al., 2015), and disrupted functioning of FFG and PHG in anxiety disorders including GAD and social anxiety disorder (SAD) (Ball et al., 2013; Frick et al., 2013; Hattingh et al., 2013; Cui et al., 2016). Specifically, the FFG is particularly associated with the processing of fearful facial and body expressions (Hadjikhani and de Gelder, 2003), whereas the PHG belongs to the paralimbic system which supports the information transition between the limbic system and the neocortex to facilitate emotion regulation (Mesulam, 2000; Kiehl, 2006). The decreased functional connectivity between the SMG and the FFG/PHG cluster in the present study, therefore, may mirror the dysfunctional attention orientation and integration of sensorimotor signals in GAD patients, which is the foundation for subsequent emotion regulation. In addition, we also observed decreased functional connectivity between SMG and the precuneus/cuneus cluster in GAD patients. The structural and functional abnormalities of precuneus and cuneus have been consistently implicated in pathological anxiety (Liao et al., 2010; Strawn et al., 2013, 2014; Wehry et al., 2015). These regions are involved in self-processing, theory of mind, and social cognition (Cavanna and Trimble, 2006; Völlm et al., 2006; Gentili et al., 2009). More importantly, previous studies have linked SMG and precuneus to the attention regulation process of emotional reactivity and regulation in both community populations and individuals with anxiety disorders (Goldin et al., 2009; Domes et al., 2010). Based on these previous findings, the reduced functional connectivity between SMG and precuneus/cuneus in the current study may reflect the aberrant sensorimotor integration and biased interpretations of social stimuli, which may result in excessive and uncontrollable worry in GAD. Taken together, this evidence implies that the SMG-based network possibly plays an important role in sensory perception and integration which facilitate social cognition and emotion regulation in GAD patients.

We also observed reduced FCS in the right SPG in adolescents with GAD. SPG is associated with top-down attention and cognitive control (Astafiev et al., 2003; Egner and Hirsch, 2005), thus the weakened FCS in this region may mirror the attentional bias toward threat in individuals with GAD as indicated by a meta-analysis (Bar-Haim et al., 2007). Furthermore, weakened functional connectivity in the SPG-based network was also found in GAD patients, including SMA and MCG. It is intriguing that SMA and MCG are not only involved in sensorimotor processing (Nachev et al., 2008; Stevens et al., 2011), but also implicated in top-down executive control on attentional and emotional process (Hopfinger et al., 2000; Nachev et al., 2008; Kalisch, 2009; Stevens et al., 2011; Kohn et al., 2014; Etkin et al., 2015). The SMA and MCG cluster in the current study also overlaps with the regions involved in cognitive reappraisal, an important process underlying successful emotion regulation (Ochsner and Gross, 2005; Kalisch, 2009). Therefore, decreased functional connectivity in the SPG-based network may imply the failure of cognitive control over salience detection and internal emotional signals, which could engender information processing bias (e.g., a preference for noticing potential threatening signals) and emotion regulation failure in GAD patients. Supporting this notion, a previous study demonstrated that GAD patients had difficulty in drawing attention away from fearful stimuli (Olatunji et al., 2011). In summary, our observations highlight the role of the SPG-based network in GAD and add evidence to the effectiveness of the treatments centered on modifying cognitive bias in GAD to some extent (e.g., cognitive-behavioral therapy).

Furthermore, functional connectivity between the SMG-based network and the SPG-based network was also reduced in the GAD patient group. Numerous studies have corroborated the role of the between-network interactions in regulating emotions (e.g., fear) (Ochsner and Gross, 2005; Delgado et al., 2008), indicating that mental disorders related to emotion regulation failure (e.g., anxiety disorders) could result from aberrant information exchange among multiple brain networks (Sylvester et al., 2012). Our observation of the decreased functional connectivity between the SMG-based network and the SPG-based network may serve as the neural representation of ineffective top-down modulation of the sensorimotor signals in GAD, which is in accordance with the characteristics of over-sensitivity to interoceptive information in anxiety disorders (Domschke et al., 2010). In summary, our findings support the multiple-networks interaction hypothesis in psychopathology, and more specifically, the notion that GAD patients have difficulty in exchanging and integrating signals among different networks (Sylvester et al., 2012).

In the GAD patient group, intra-network connectivity of the SMG-based network and inter-network connectivity between the SMG-based network and the SPG-based network were positively correlated with the STAI score and the trait score of STAI, respectively. However, the two neuroimaging indexes were lower while the two clinical scores were higher in the GAD patient group compared with HCs. Indeed, this counterintuitive brain-clinical association has been widely found in mental disorders, including GAD (Etkin et al., 2009), posttraumatic stress disorder (PTSD) (Villarreal et al., 2004; Kim et al., 2007), attention deficit hyperactivity disorder (ADHD) (Suskauer et al., 2008; Tao et al., 2017), and schizophrenia (Gur et al., 1998). Although the understanding of the paradoxical brain-clinical correlation in clinical neuroscience is still limited, we tentatively provided a possible explanation in the current study. Given the reduced functional connectivity in the SMG-based network and between the SMG-based network and the SPG-based network, GAD patients may try to increase functional connectivity to become “normal” by using certain emotion regulation strategies. However, these strategies are ineffective, or even increase anxiety. One example of these deleterious strategies is the positive beliefs on worry, which has been frequently employed by GAD patients (Borkovec et al., 1999). These individuals with GAD tend to believe that worry can help them cope with adversity more successfully (Llera and Newman, 2010), but it ends up reinforcing worry, and this creates a vicious cycle. In other words, the positive correlations between functional connectivity and anxiety severity may reflect the maladaptive strategies employed by GAD patients to deal with negative emotions. Notably, these hypotheses were not directly tested in the current study, thus should be treated with caution and tested in future studies.

Furthermore, the linear regression model demonstrated that 25.5% variance of STAI and 39.5% variance of the trait score of STAI could be explained by functional connectivity. These results imply that the functional connectivity measures can serve as biomarkers for GAD. It is interesting that none of the three predictors (intra-network connectivity of the SMG-based network, the SPG-based network, and inter-network connectivity between the SMG-based network and the SPG-based network) managed to predict clinical symptoms independently, while the combined regression models did successfully explain the symptom variance. These findings indicate that the interactions among networks are more crucial than any individual network to predict GAD symptoms. Therefore, they further support the advantages of understanding the pathological mechanisms of GAD by investigating multiple subnetworks.

Notably, some GAD-related regional alterations demonstrated in previous work were not identified in the present study [e.g., amygdala, hippocampus, ventral gyrus of anterior cingulate cortex (vgACC), and DLPFC]. This discrepancy may result from different methodologies. Previous studies basically hypothesized specific regional disruptions in GAD and predefined these regions as the seed regions (e.g., Etkin et al., 2009; Chen and Etkin, 2013; Andreescu et al., 2014; Li et al., 2016). In the current study, however, we used a whole-brain voxel-wise data-driven approach, which gives a bigger picture of the aberrant functional architecture of GAD. Another possibility is the heterogeneity of the GAD sample. Most previous R-fMRI studies examining the abnormal functional architecture of GAD recruited adult patients (Etkin et al., 2009; Etkin and Schatzberg, 2011; Chen and Etkin, 2013; Andreescu et al., 2014; Liu et al., 2015; Cui et al., 2016; Li et al., 2016). In the few studies that centered on adolescent GAD patients, medication use could be a potential confound (Strawn et al., 2012; Liu et al., 2015).

**FIGURE 4:**
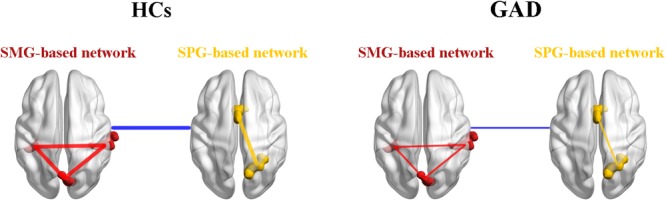
A theoretical model of the disrupted functional networks in GAD. Intra-network connectivity is depicted by red lines in the SMG-based network and yellow lines in the SPG-based network, while inter-network connectivity is depicted by blue lines. The whole model illustrates that intra-network connectivity of the SMG-based network, the SPG-based network, and inter-network connectivity between the SMG-based network and the SPG-based network were decreased in the GAD patient group. HCs, healthy controls; GAD, generalized anxiety disorder; SMG, supramarginal gyrus; SPG, superior parietal gyrus.

Based on our findings, we proposed a theoretical model of GAD (Figure 4). We suggest that the decreased functional connectivity within the SMG-based network, the SPG-based network, and between the SMG-based network and the SPG-based network could be the underlying psychopathological mechanisms in the functional brain network in this disorder. This model will need further validation by future research, which will contribute to the understanding of GAD.

Several limitations and methodological issues should be considered further. First, the sample size of the current study is relatively small, which decreases the statistical power. Future research recruiting more participants would be quite valuable and informative. Second, although one possibility of the counterintuitive brain-behavior association was provided in the current study, it still remains untested. Future studies are needed to test this interpretation and also consider other potential explanations. Finally, the abnormal functional connectivity maps of GAD are probably associated with structural characteristics (e.g., cortical thickness, regional volumes). Future research is recommended to investigate structural and functional coupling in GAD, which could help develop a more sophisticated pathological model of this disorder.

## Conclusion

The present study used resting-state fMRI to explore the functional connectivity alterations in neural subnetworks of adolescents with GAD, and found decreased functional connectivity in the SMG-based network, the SPG-based network, and between the SMG-based network and the SPG-based network in this disorder. Our findings highlight the aberrant functional architecture in the SMG-based network and the SPG-based network in GAD, providing novel insights into the pathophysiological mechanisms of this disorder.

## Author Contributions

FY, YZ, LL, LS, and ZD designed the study. TZ and ML acquired the data, which LF, JM, and FY analyzed. LF, ZD, YL, JM, and YW wrote the article, which all authors reviewed and approved for publication.

## Conflict of Interest Statement

The authors declare that the research was conducted in the absence of any commercial or financial relationships that could be construed as a potential conflict of interest.

## Footnotes

1 http://www.fil.ion.ucl.ac.uk/spm

2 http://www.nitrc.org/projects/bnv/

## References

[B1] American Psychiatric Association (2013). *Diagnostic and Statistical Manual of Mental Disorders (DSM-5)* 5th Edn. Arlington, VA: American Psychiatric Publishing 10.1176/appi.books.9780890425596

[B2] Andreescu C. Sheu L. K. Tudorascu D. Walker S. Aizenstein H. (2014). The ages of anxiety—differences across the lifespan in the default mode network functional connectivity in generalized anxiety disorder. *Int. J. Geriatr. Psychiatry* 29 704–712. 10.1002/gps.4051 24254806 PMC4028428

[B3] Astafiev S. V. Shulman G. L. Stanley C. M. Snyder A. Z. Van Essen D. C. Corbetta M. (2003). Functional organization of human intraparietal and frontal cortex for attending, looking, and pointing. *J. Neurosci.* 23 4689–4699. 10.1523/JNEUROSCI.23-11-04689.2003 12805308 PMC6740811

[B4] Ball T. M. Ramsawh H. J. Campbell-Sills L. Paulus M. P. Stein M. B. (2013). Prefrontal dysfunction during emotion regulation in generalized anxiety and panic disorders. *Psychol. Med.* 43 1475–1486. 10.1017/S0033291712002383 23111120 PMC4308620

[B5] Bar-Haim Y. Lamy D. Pergamin L. Bakermans-Kranenburg M. J. van IJzendoorn M. H. (2007). Threat-related attentional bias in anxious and nonanxious individuals: a meta-analytic study. *Psychol. Bull.* 133 1–24. 10.1037/0033-2909.133.1.1 17201568

[B6] Birmaher B. Brent D. A. Chiappetta L. Bridge J. Monga S. Baugher M. (1999). Psychometric properties of the screen for child anxiety related emotional disorders (SCARED): a replication study. *J. Am. Acad. Child Adolesc. Psychiatry* 38 1230–1236. 10.1097/00004583-199910000-00011 10517055

[B7] Biswal B. Mennes M. Zuo X. Gohel S. Kelly C. Smith S. M. (2010). Toward discovery science of human brain function. *Proc. Natl. Acad. Sci. U.S.A.* 107 4734–4739. 10.1073/pnas.0911855107 20176931 PMC2842060

[B8] Biswal B. Yetkin F. Z. Haughton V. M. Hyde J. S. (1995). Functional connectivity in the motor cortex of resting human brain using echo-planar MRI. *Magn. Reson. Med.* 34 537–541. 10.1002/mrm.1910340409 8524021

[B9] Borkovec T. D. Hazlett-Stevens H. Diaz M. L. (1999). The role of positive beliefs about worry in generalized anxiety disorder and its treatment. *Clin. Psychol. Psychother.* 6 126–138. 10.1002/(SICI)1099-0879(199905)6:2<126::AID-CPP193<3.0.CO;2-M

[B10] Buckner R. L. Sepulcre J. Talukdar T. Krienen F. M. Liu H. Hedden T. (2009). Cortical hubs revealed by intrinsic functional connectivity: mapping, assessment of stability, and relation to Alzheimer’s disease. *J. Neurosci.* 29 1860–1873. 10.1523/JNEUROSCI.5062-08.2009 19211893 PMC2750039

[B11] Bullmore E. Sporns O. (2009). Complex brain networks: graph theoretical analysis of structural and functional systems. *Nat. Rev. Neurosci.* 10 186–198. 10.1038/nrn257 19190637

[B12] Cavanna A. E. Trimble M. R. (2006). The precuneus: a review of its functional anatomy and behavioural correlates. *Brain* 129 564–583. 10.1093/brain/awl004 16399806

[B13] Cavedo E. Chiesa P. Houot M. Ferretti M. Grothe M. Teipel S. (2018). Sex differences in functional and molecular neuroimaging biomarkers of Alzheimer’s disease in cognitively normal older adults with subjective memory complaints. *Alzheimers Dement.* 14 1204–1215. 10.1016/j.jalz.2018.05.014 30201102

[B14] Chen A. C. Etkin A. (2013). Hippocampal network connectivity and activation differentiates post-traumatic stress disorder from generalized anxiety disorder. *Neuropsychopharmacoloy* 38 1889–1898. 10.1038/npp.2013.122 23673864 PMC3746693

[B15] Cole D. M. Smith S. M. Beckmann C. F. (2010). Advances and pitfalls in the analysis and interpretation of resting-state FMRI data. *Front. Syst. Neurosci.* 4:8. 10.3389/fnsys.2010.00008 20407579 PMC2854531

[B16] Copeland W. E. Shanahan L. Worthman C. Angold A. Costello E. J. (2012). Generalized anxiety and C-reactive protein levels: a prospective, longitudinal analysis. *Psychol. Med.* 42 2641–2650. 10.1017/S0033291712000554 22716910 PMC3449031

[B17] Costello E. J. Mustillo S. Erkanli A. Keeler G. Angold A. (2003). Prevalence and development of psychiatric disorders in childhood and adolescence. *Arch. Gen. Psychiatry* 60 837–844. 10.1001/archpsyc.60.8.837 12912767

[B18] Cox R. W. (2012). AFNI: what a long strange trip it’s been. *Neuroimage* 62 743–747. 10.1016/j.neuroimage.2011.08.056 21889996 PMC3246532

[B19] Cui H. Zhang J. Liu Y. Li Q. Li H. Zhang L. (2016). Differential alterations of resting-state functional connectivity in generalized anxiety disorder and panic disorder. *Hum. Brain Mapp.* 37 1459–1473. 10.1002/hbm.23113 26800659 PMC6867341

[B20] Dai Z. Yan C. Li K. Wang Z. Wang J. Cao M. (2015). Identifying and mapping connectivity patterns of brain network hubs in Alzheimer’s Disease. *Cereb. Cortex* 25 3723–3742. 10.1093/cercor/bhu246 25331602

[B21] de Reus M. A. van den Heuvel M. P. (2013). The parcellation-based connectome: limitations and extensions. *Neuroimage* 80 397–404. 10.1016/j.neuroimage.2013.03.053 23558097

[B22] Delgado M. R. Nearing K. I. LeDoux J. E. Phelps E. A. (2008). Neural circuitry underlying the regulation of conditioned fear and its relation to extinction. *Neuron* 59 829–838. 10.1016/j.neuron.2008.06.029 18786365 PMC3061554

[B23] Domes G. Schulze L. Böttger M. Grossmann A. Hauenstein K. Wirtz P. H. (2010). The neural correlates of sex differences in emotional reactivity and emotion regulation. *Hum. Brain Mapp.* 31 758–769. 10.1002/hbm.20903 19957268 PMC6871188

[B24] Domschke K. Stevens S. Pfleiderer B. Gerlach A. (2010). Interoceptive sensitivity in anxiety and anxiety disorders: an overview and integration of neurobiological findings. *Clin. Psychol. Rev.* 30 1–11. 10.1016/j.cpr.2009.08.008 19751958

[B25] Egner T. Hirsch J. (2005). The neural correlates and functional integration of cognitive control in a Stroop task. *Neuroimage* 24 539–547. 10.1016/j.neuroimage.2004.09.00 15627596

[B26] Enrici I. Adenzato M. Cappa S. Bara B. G. Tettamanti M. (2011). Intention processing in communication: a common brain network for language and gestures. *J. Cogn. Neurosci.* 23 2415–2431. 10.1162/jocn.2010.21594 20954937

[B27] Etkin A. Büchel C. Gross J. J. (2015). The neural bases of emotion regulation. *Nat. Rev. Neurosci.* 16 693–700. 10.1038/nrn4044 26481098

[B28] Etkin A. Prater K. E. Schatzberg A. F. Menon V. Greicius M. D. (2009). Disrupted amygdalar subregion functional connectivity and evidence of a compensatory network in generalized anxiety disorder. *Arch. Gen. Psychiatry* 66 1361–1372. 10.1001/archgenpsychiatry.2009.104 19996041 PMC12553334

[B29] Etkin A. Schatzberg A. F. (2011). Common abnormalities and disorder-specific compensation during implicit regulation of emotional processing in generalized anxiety and major depressive disorders. *Am. J. Psychiatry* 168 968–978. 10.1176/appi.ajp.2011.10091290 21632648

[B30] Frick A. Howner K. Fischer H. Kristiansson M. Furmark T. (2013). Altered fusiform connectivity during processing of fearful faces in social anxiety disorder. *Transl. Psychiatry* 3:e312. 10.1038/tp.2013.85 24105443 PMC3818016

[B31] Fritz C. O. Morris P. E. Richler J. J. (2012). Effect size estimates: current use, calculations, and interpretation. *J. Exp. Psychol. Gen.* 141 2–18. 10.1037/a002433 21823805

[B32] Gentili C. Ricciardi E. Gobbini M. I. Santarelli M. F. Haxby J. V. Pietrini P. (2009). Beyond amygdala: default mode network activity differs between patients with social phobia and healthy controls. *Brain Res. Bull.* 79 409–413. 10.1016/j.brainresbull.2009.02.002 19559343

[B33] Goldin P. R. Manber T. Hakimi S. Canli T. Gross J. J. (2009). Neural bases of social anxiety disorder: emotional reactivity and cognitive regulation during social and physical threat. *Arch. Gen. Psychiatry* 66 170–180. 10.1001/archgenpsychiatry.2008.525 19188539 PMC4142809

[B34] Gur R. E. Cowell P. Turetsky B. I. Gallacher F. Cannon T. Bilker W. (1998). A follow-up magnetic resonance imaging study of schizophrenia: relationship of neuroanatomical changes to clinical and neurobehavioral measures. *Arch. Gen. Psychiatry* 55 145–152. 10.1001/archpsyc.55.2.145 9477928

[B35] Hadjikhani N. de Gelder B. (2003). Seeing fearful body expressions activates the fusiform cortex and amygdala. *Curr. Biol.* 13 2201–2205. 10.1016/j.cub.2003.11.049 14680638

[B36] Hattingh C. J. Ipser J. Tromp S. Syal S. Lochner C. Brooks S. J. B. (2013). Functional magnetic resonance imaging during emotion recognition in social anxiety disorder: an activation likelihood meta-analysis. *Front. Hum. Neurosci.* 6:347. 10.3389/fnhum.2012.00347 23335892 PMC3547329

[B37] Hernández S. E. Barros-Loscertales A. Xiao Y. González-Mora J. L. Rubia K. (2018). Gray matter and functional connectivity in anterior cingulate cortex are associated with the state of mental silence during sahaja yoga meditation. *Neuroscience* 371 395–406. 10.1016/j.neuroscience.2017.12.017 29275207

[B38] Hopfinger J. B. Buonocore M. H. Mangun G. R. (2000). The neural mechanisms of top-down attentional control. *Nat. Neurosci.* 3 284–291. 10.1038/72999 10700262

[B39] Jenkinson M. Bannister P. Brady M. Smith S. (2002). Improved optimization for the robust and accurate linear registration and motion correction of brain images. *Neuroimage* 17 825–841. 10.1006/nimg.2002.1132 12377157

[B40] Kalisch R. (2009). The functional neuroanatomy of reappraisal: time matters. *Neurosci. Biobehav. Rev.* 33 1215–1226. 10.1016/j.neubiorev.2009.06.003 19539645

[B41] Kaufman J. Birmaher B. Brent D. Rao U. Flynn C. Moreci P. (1997). Schedule for affective disorders and schizophrenia for school-age children-present and lifetime version (K-SADS-PL): initial reliability and validity data. *J. Am. Acad. Child Adolesc. Psychiatry* 36 980–988. 10.1097/00004583-199707000-00021 9204677

[B42] Kelly C. Biswal B. Craddock R. C. Castellanos F. X. Milham M. P. (2012). Characterizing variation in the functional connectome: promise and pitfalls. *Trends Cogn. Sci.* 16 181–188. 10.1016/j.tics.2012.02.001 22341211 PMC3882689

[B43] Kiehl K. A. (2006). A cognitive neuroscience perspective on psychopathy: evidence for paralimbic system dysfunction. *Psychiatry Res.* 142 107–128. 10.1016/j.psychres.2005.09.013 16712954 PMC2765815

[B44] Kim S. J. Lyoo I. K. Lee Y. S. Kim J. Sim M. E. Bae S. J. (2007). Decreased cerebral blood flow of thalamus in PTSD patients as a strategy to reduce re-experience symptoms. *Acta Psychiatr. Scand.* 116 145–153. 10.1111/j.1600-0447.2006.00952.x 17650277

[B45] Kohn N. Eickhoff S. B. Scheller M. Laird A. R. Fox P. T. Habel U. (2014). Neural network of cognitive emotion regulation—an ALE meta-analysis and MACM analysis. *Neuroimage* 87 345–355. 10.1016/j.neuroimage.2013.11.001 24220041 PMC4801480

[B46] Li W. Cui H. Zhu Z. Kong L. Guo Q. Zhu Y. (2016). Aberrant functional connectivity between the amygdala and the temporal pole in drug-free generalized anxiety disorder. *Front. Hum. Neurosci.* 10:549. 10.3389/fnhum.2016.00549 27867352 PMC5095112

[B47] Liang X. Zou Q. He Y. Yang Y. (2013). Coupling of functional connectivity and regional cerebral blood flow reveals a physiological basis for network hubs of the human brain. *Proc. Natl. Acad. Sci. U.S.A.* 110 1929–1934. 10.1073/pnas.1214900110 23319644 PMC3562840

[B48] Liao M. Yang F. Zhang Y. He Z. Su L. Li L. (2014). Lack of gender effects on gray matter volumes in adolescent generalized anxiety disorder. *J. Affect. Disord.* 155 278–282. 10.1016/j.jad.2013.10.049 24262640

[B49] Liao W. Chen H. Feng Y. Mantini D. Gentili C. Pan Z. (2010). Selective aberrant functional connectivity of resting state networks in social anxiety disorder. *Neuroimage* 52 1549–1558. 10.1016/j.neuroimage.2010.05.010 20470894

[B50] Liu J. Xia M. Dai Z. Wang X. Liao X. Bi Y. (2016). Intrinsic brain hub connectivity underlies individual differences in spatial working memory. *Cereb. Cortex* 27 5496–5508. 10.1093/cercor/bhw317 28334075

[B51] Liu W. Yin D. Cheng W. Fan M. You M. Men W. (2015). Abnormal functional connectivity of the amygdala-based network in resting-state FMRI in adolescents with generalized anxiety disorder. *Med. Sci. Mon. Int. Med. J. Exp. Clin. Res.* 21:459. 10.12659/MSM.893373 25673008 PMC4335563

[B52] Llera S. J. Newman M. G. (2010). Effects of worry on physiological and subjective reactivity to emotional stimuli in generalized anxiety disorder and nonanxious control participants. *Emotion* 10 640–650. 10.1037/a0019351 21038947

[B53] Lopez C. Blanke O. (2011). The thalamocortical vestibular system in animals and humans. *Brain Res. Rev.* 67 119–146. 10.1016/j.brainresrev.2010.12.0 21223979

[B54] Merikangas K. R. He J. Burstein M. Swanson S. A. Avenevoli S. Cui L. (2010). Lifetime prevalence of mental disorders in U.S. adolescents: results from the national comorbidity survey replication–Adolescent supplement (NCS-A). *J. Am. Acad. Child. Adolesc. Psychiatry* 49 980–989. 10.1016/j.jaac.2010.05.017 20855043 PMC2946114

[B55] Mesulam M. M. (2000). *Principles of Behavioral and Cognitive Neurology* 2nd Edn. New York, NY: Oxford University Press.

[B56] Modi S. Kumar M. Kumar P. Khushu S. (2015). Aberrant functional connectivity of resting state networks associated with trait anxiety. *Psychiatry Res. Neuroimaging* 234 25–34. 10.1016/j.pscychresns.2015.07.006 26385540

[B57] Murphy K. Birn R. M. Handwerker D. A. Jones T. B. Bandettini P. A. (2009). The impact of global signal regression on resting state correlations: are anti-correlated networks introduced? *Neuroimage* 44 893–905. 10.1016/j.neuroimage.2008.09.036 18976716 PMC2750906

[B58] Nachev P. Kennard C. Husain M. (2008). Functional role of the supplementary and pre-supplementary motor areas. *Nat. Rev. Neurosci.* 9 856–869. 10.1038/nrn2478 18843271

[B59] Ochsner K. N. Gross J. J. (2005). The cognitive control of emotion. *Trends Cogn. Sci.* 9 242–249. 10.1016/j.tics.2005.03.010 15866151

[B60] Olatunji B. O. Ciesielski B. G. Armstrong T. Zhao M. Zald D. H. (2011). Making something out of nothing: neutral content modulates attention in generalized anxiety disorder. *Depress Anxiety* 28 427–434. 10.1002/da.20806 21449004 PMC5487026

[B61] Power J. D. Barnes K. A. Snyder A. Z. Schlaggar B. L. Petersen S. E. (2012). Spurious but systematic correlations in functional connectivity MRI networks arise from subject motion. *Neuroimage* 59 2142–2154. 10.1016/j.neuroimage.2011.10.018 22019881 PMC3254728

[B62] Roy A. K. Fudge J. L. Kelly C. Perry J. S. Daniele T. Carlisi C. (2013). Intrinsic functional connectivity of amygdala-based networks in adolescent generalized anxiety disorder. *J. Am. Acad. Child Adolesc. Psychiatry* 52 290–299. 10.1016/j.jaac.2012.12.010 23452685 PMC3760686

[B63] Rubinov M. Sporns O. (2010). Complex network measures of brain connectivity: uses and interpretations. *Neuroimage* 52 1059–1069. 10.1016/j.neuroimage.2009.10.003 19819337

[B64] Satterthwaite T. D. Wolf D. H. Loughead J. Ruparel K. Elliott M. A. Hakonarson H. (2012). Impact of in-scanner head motion on multiple measures of functional connectivity: relevance for studies of neurodevelopment in youth. *Neuroimage* 60 623–632. 10.1016/j.neuroimage.2011.12.063 22233733 PMC3746318

[B65] Silani G. Lamm C. Ruff C. C. Singer T. (2013). Right supramarginal gyrus is crucial to overcome emotional egocentricity bias in social judgments. *J. Neurosci.* 33 15466–15476. 10.1523/JNEUROSCI.1488-13.2013 24068815 PMC6618458

[B66] Song X. W. Dong Z. Y. Long X. Y. Li S. F. Zuo X. N. Zhu C. Z. (2011). REST: a toolkit for resting-state functional magnetic resonance imaging data processing. *PLoS One* 6:e25031. 10.1371/journal.pone.0025031 21949842 PMC3176805

[B67] Stevens F. L. Hurley R. A. Taber K. H. (2011). Anterior cingulate cortex: unique role in cognition and emotion. *J. Neuropsychiatry Clin. Neurosci.* 23 121–125. 10.1176/jnp.23.2.jnp121 21677237

[B68] Strawn J. R. Bitter S. M. Weber W. A. Chu W. Whitsel R. M. Adler C. (2012). Neurocircuitry of generalized anxiety disorder in adolescents: a pilot functional neuroimaging and functional connectivity study. *Depress Anxiety* 29 939–947. 10.1002/da.21961 22628125

[B69] Strawn J. R. Dominick K. C. Patino L. R. Doyle C. D. Picard L. S. Phan K. L. (2014). Neurobiology of pediatric anxiety disorders. *Curr. Behav. Neurosci. Rep.* 1 154–160. 10.1007/s40473-014-0014-1 25745592 PMC4347469

[B70] Strawn J. R. Wehry A. M. Chu W. J. Adler C. M. Eliassen J. C. Cerullo M. A. (2013). Neuroanatomic abnormalities in adolescents with generalized anxiety disorder: a voxel-based morphometry study. *Depress. Anxiety* 30 842–848. 10.1002/da.22089 23495075

[B71] Su L. Wang K. Fan F. Su Y. Gao X. (2008). Reliability and validity of the screen for child anxiety related emotional disorders (SCARED) in Chinese children. *J. Anxiety Disord.* 22 612–621. 10.1016/j.janxdis.2007.05.011 17628391

[B72] Suskauer S. J. Simmonds D. J. Caffo B. S. Denckla M. B. Pekar J. J. Mostofsky S. H. (2008). fMRI of intrasubject variability in ADHD: anomalous premotor activity with prefrontal compensation. *J. Am. Acad. Child Adolesc. Psychiatry* 47 1141–1150. 10.1097/CHI.0b013e3181825b1f 18724253 PMC3932630

[B73] Sylvester C. M. Corbetta M. Raichle M. E. Rodebaugh T. L. Schlaggar B. L. Sheline Y. I. (2012). Functional network dysfunction in anxiety and anxiety disorders. *Trends Neurosci.* 35 527–535. 10.1016/j.tins.2012.04.012 22658924 PMC3432139

[B74] Takeuchi H. Taki Y. Nouchi R. Yokoyama R. Kotozaki Y. Nakagawa S. (2017). Regional homogeneity, resting-state functional connectivity and amplitude of low frequency fluctuation associated with creativity measured by divergent thinking in a sex-specific manner. *Neuroimage* 152 258–269. 10.1016/j.neuroimage.2017.02.079 28257930

[B75] Tao J. Jiang X. Wang X. Liu H. Qian A. Yang C. (2017). Disrupted control-related functional brain networks in drug-naive children with attention-deficit/hyperactivity disorder. *Front. Psychiatry* 8:246. 10.3389/fpsyt.2017.00246 29209238 PMC5702526

[B76] Van Dijk K. R. Sabuncu M. R. Buckner R. L. (2012). The influence of head motion on intrinsic functional connectivity MRI. *Neuroimage* 59 431–438. 10.1016/j.neuroimage.2011.07.044 21810475 PMC3683830

[B77] Villarreal G. Hamilton D. A. Graham D. P. Driscoll I. Qualls C. Petropoulos H. (2004). Reduced area of the corpus callosum in posttraumatic stress disorder. *Psychiatry Res. Neuroimaging* 131 227–235. 10.1016/j.pscychresns.2004.05.002 15465292

[B78] Völlm B. A. Taylor A. N. Richardson P. Corcoran R. Stirling J. McKie S. (2006). Neuronal correlates of theory of mind and empathy: a functional magnetic resonance imaging study in a nonverbal task. *Neuroimage* 29 90–98. 10.1016/j.neuroimage.2005.07.022 16122944

[B79] Wehry A. M. Beesdo-Baum K. Hennelly M. M. Connolly S. D. Strawn J. R. (2015). Assessment and treatment of anxiety disorders in children and adolescents. *Curr. Psychiatry Rep.* 17:52. 10.1007/s11920-015-0591-z 25980507 PMC4480225

[B80] Xia M. Wang J. He Y. (2013). BrainNet Viewer: a network visualization tool for human brain connectomics. *PLoS One* 8:e68910. 10.1371/journal.pone.0068910 23861951 PMC3701683

[B81] Yan C. Zang Y. (2010). DPARSF: a MATLAB toolbox for “Pipeline” data analysis of resting-state fMRI. *Front. Syst. Neurosci.* 4:13 10.3389/fnsys.2010.00013 PMC2889691 20577591

[B82] Yan C. G. Wang X. D. Zuo X. N. Zang Y. F. (2016). DPABI: data processing & analysis for (resting-state) brain imaging. *Neuroinformatics* 14 339–351. 10.1007/s12021-016-9299-4 27075850

[B83] Yao Z. Liao M. Hu T. Zhang Z. Zhao Y. Zheng F. (2017). An effective method to identify adolescent generalized anxiety disorder by temporal features of dynamic functional connectivity. *Front. Hum. Neurosci.* 11:492. 10.3389/fnhum.2017.00492 29081741 PMC5645525

[B84] Zhang Z. Liao M. Yao Z. Hu B. Xie Y. Zheng W. (2017). Frequency-specific functional connectivity density as an effective biomarker for adolescent generalized anxiety disorder. *Front. Hum. Neurosci.* 11:549. 10.3389/fnhum.2017.00549 29259549 PMC5723402

[B85] Zu Eulenburg P. Caspers S. Roski C. Eickhoff S. B. (2012). Meta-analytical definition and functional connectivity of the human vestibular cortex. *Neuroimage* 60 162–169. 10.1016/j.neuroimage.2011.12.03 22209784

