# Supplementary material for: Decreased Intrinsic Functional Connectivity in First-Episode, Drug-Naive Adolescents With Generalized Anxiety Disorder
Source: Front Hum Neurosci. 2019 Jan 11;12:539. doi: 10.3389/fnhum.2018.00539 (PMC6337053; doi:10.3389/fnhum.2018.00539)
Supplement: Supplementary file 1 [file Data_Sheet_1.docx]

Supplementary Material

**Decreased Intrinsic Functional Connectivity in First-Episode, Drug-Naive Adolescents with Generalized Anxiety Disorder**

Fan Yang^#^, Linlin Fan^#^, Tianyi Zhai, Ying Lin, Yuyin Wang, Junji Ma, Mei Liao, Yan Zhang, Lingjiang Li, Linyan Su^*^, Zhengjia Dai^*^

^#^Fan Yang and Linlin Fan contributed equally to this work.

**^*^Corresponding to:**

Zhengjia Dai

[daizhengj@mail.sysu.edu.cn](mailto:daizhengj@mail.sysu.edu.cn)

Linyan Su

[xysulinyan@126.com](mailto:xysulinyan@126.com)

# Supplementary Figures


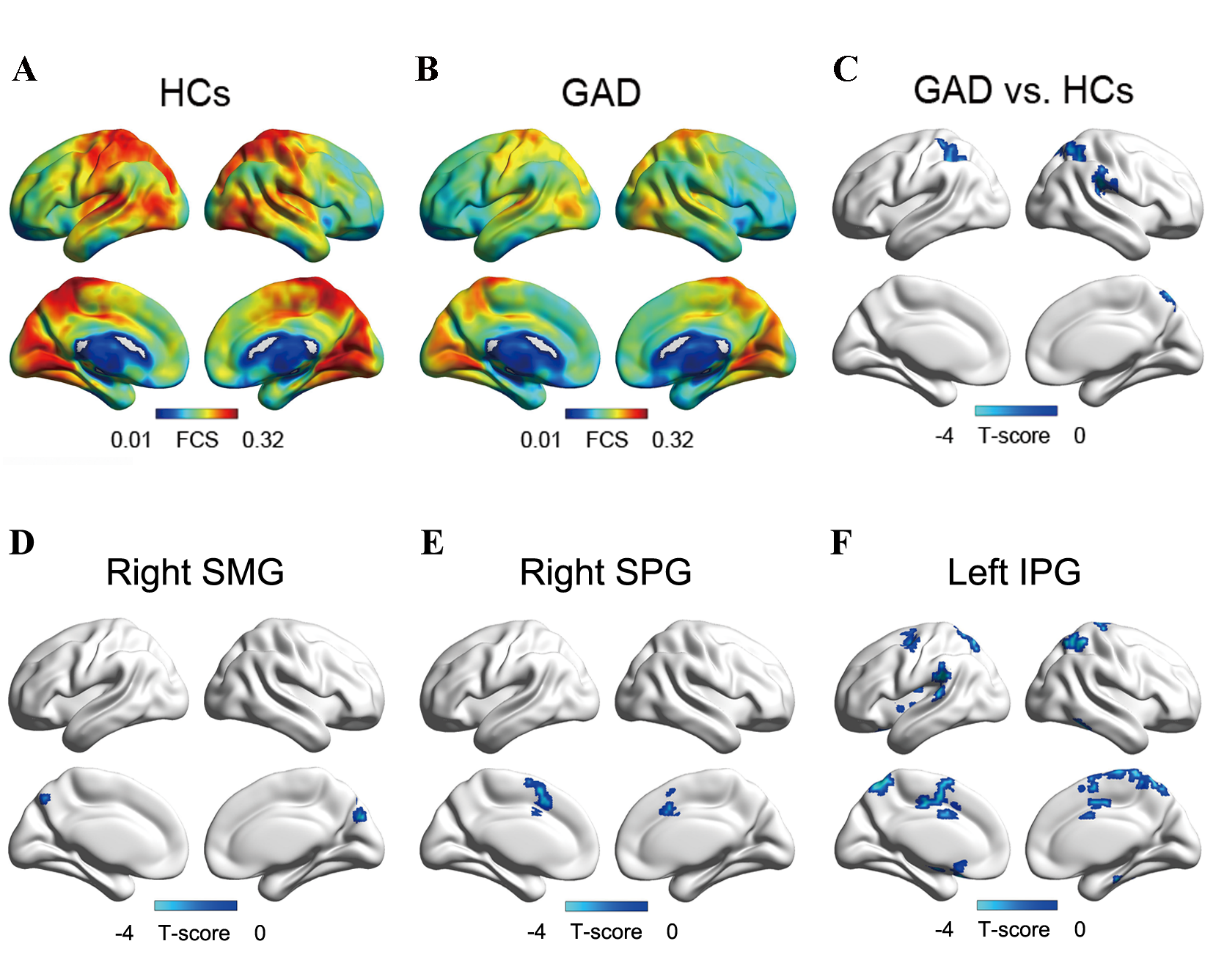


**Figure S1.** FCS maps of the HCs group (A) and the GAD patient group (B), and between-group differences of FCS (C), of FC maps of right SMG (D), right SPG (E), and left IPG (F) after scrubbing. Significance threshold of FCS was set at *p* < 0.01 with cluster size of 110 voxels, corresponding to a corrected *p* < 0.05, and that of FC was set at *p* < 0.001 with cluster size of 53 voxels for right SMG as ROI, 47 voxels for right SPG as ROI, and 50 voxels for left IPG as ROI, all corresponding to corrected *p*s < 0.01. HCs, healthy controls; GAD, generalized anxiety disorder; SMG, supramarginal gyrus; SPG, superior parietal gyrus; IPG, inferior parietal gyrus.
